# Immediate Effects of Extracorporeal Shock Wave Therapy in Fascial Fibroblasts: An In Vitro Study

**DOI:** 10.3390/biomedicines10071732

**Published:** 2022-07-18

**Authors:** Carmelo Pirri, Caterina Fede, Lucia Petrelli, Enrico De Rose, Carlo Biz, Diego Guidolin, Raffaele De Caro, Carla Stecco

**Affiliations:** 1 Institute of Humana Anatomy, Department of Neurosciences, University of Padova, 35121 Padua, Italy; caterina.fede@unipd.it (C.F.); lucia.petrelli@unipd.it (L.P.); enrico.derose@studenti.unipd.it (E.D.R.); diego.guidolin@unipd.it (D.G.); rdecaro@unipd.it (R.D.C.); 2 Orthopedics and Orthopedic Oncology, Department of Surgery, Oncology and Gastroenterology (DiSCOG), University of Padua, 35128 Padua, Italy; carlo.biz@unipd.it

**Keywords:** fascia, hyaluronan, vesicles, myofascial pain, focused extracorporeal shock waves, 3D printer

## Abstract

Extracorporeal shock waves (ESWs) are used in the treatment of soft tissue injuries, but their role in the treatment of myofascial pain has not yet been demonstrated. The aim of this study was to investigate changes in cell biology of fibroblasts derived from deep/muscular fascia following treatment with ESWs. Primary fascial fibroblasts were collected from small samples of human fascia lata of the thigh of three volunteer patients (two men, one woman) during orthopedic surgery, and put in culture. These cells were exposed to 100 impulses of 0.05 mJ/mm^2^ with a frequency of 2.5 Hz, using 3D-printed support. This study demonstrated for the first time that ESWs can lead to in vitro production of hyaluronan-rich vesicles immediately after the treatment. At 1, 4, and 24 h after treatment, Alcian blue and Toluidine blue staining; immunocytochemistry to detect hyaluronic acid binding protein (HABP), collagen I, and collagen III; and transmission electron microscopy demonstrated that these vesicles are rich in hyaluronan and collagen I and III. The diameter of these vesicles was assessed, highlighting a small size at 1 h after ESW treatment, whereas at 4 and 24 h, they had an increase in the size. Particularly evident was the release of hyaluronan-rich vesicles, collagen-I, and collagen-III starting at 1 h, with an increase at 4 h and maintenance by 24 h. These in vitro data indicate that fascial cells respond to ESW treatment by regulating and remodeling the formation of extracellular matrix.

## 1. Introduction

Extracorporeal shockwaves (ESWs) are acoustic waves that induce a mechanical wave that passes through the cell compartment by cavitational effects, where cellular reply is proportional to the energy used [1]. The initial use of ESWs in medicine was in kidney stone fragmentation, but their application was quickly expanded to a variety of pathologies of the musculoskeletal system, such as the treatment of non-unions, tendinopathies, and soft tissue disorders [2,3,4]. A recent systematic review [5] revealed 100 studies that addressed the effects of ESWs on bone and cartilage tissue [6,7,8], 39 studies about the effects of ESWs on tenocytes or scar tissue [9,10,11], and 42 studies on muscle and nerve tissue [12,13,14]. No studies are present about the effects of ESWs on fascial tissues, nor if myofascial pain is considered a promising indication for their use [15]. Myofascial pain (MP) is a musculoskeletal disorder with some phenomena that range from acute pain to decreased range of motion and weakness [16]. Currently, there is much evidence to suggest that fasciae are highly innerved, both with sensitive and autonomic nerve fibers, and that fascial alterations can play a key role in pain generation and, more generally, in myofascial pain [17].

Although ESWs are already widely used in clinical practice, the mechanism of action underlying their biological activities is still not fully understood. A wide variety of effects at the molecular and cellular levels have been described to date, whereby the effects of ESWs differ in each case from the tissue treated [5,18,19,20,21,22]. From the review conducted by Wuerfel [5], it appears that the more demonstrated effects of ESWs are the increase of cell proliferations of both progenitor and differentiated cells, the ability to mimic the effect of capsaicin by reducing substance-P concentration and of botulinum toxin by destroying endplates in the neuromuscular junction, and the modulation of the releases of various cytokines and collagen type I fibers. From this review, it is clear that different tissues respond to the same mechanical stimulus in different ways, and consequently it is not correct to automatically translate the data of the research on tenocytes and skin fibroblasts to fascial tissue. Fasciae are formed both by a fibrotic component that has many aspects in common with the tendons and a loose component that is rich in water and hyaluronan [23]. Collagen type 1 (COL I) is the major collagen constituent in the fasciae, while collagen type 3 (COL III) has been shown to regulate the initial fibril assembly, playing a crucial role in the early stage of injury repair [24]. Fascial fibroblasts respond to various microenvironmental stimuli. Some studies have evaluated the reply of the fascial fibroblast to hormonal [25] or pharmacological [26] stimuli. Mechano-transduction, i.e., the process that converts physical force into a biochemical signal and subsequently integrates into a cellular reply [27,28], is of growing interest due to many extracorporeal therapeutic devices that use physical energies. The bio-responses to physical energy that have been observed are an increase in cell proliferation and synthesis and remodeling of the extracellular matrix [29].

Furthermore, different hypotheses have been proposed on the molecular mechanisms by which ESW treatment alleviates the pain in the musculoskeletal system [30], but so far, only the nerve component without the connective component has been considered [30]. However, Pavan et al. [31], in considering the complex structure of deep fascia, reported that in myofascial pain, deep fascia could be subjected to two different kinds of alteration: (1) damage of loose component (rich in water and HA), affecting the sliding system between different layers and (2) damage of fibrous component, affecting the capacity of force transmission.

Therefore, the main aim of the current study was to examine cellular and ECM changes of fascial tissue induced by ESWT to reveal the short-term mechanisms underlying the beneficial effect of ESWT. To achieve this goal, we decided to perform an in vitro study on the fibroblasts isolated from fascia lata to evaluate if ESWT is able to stimulate fascial fibroblasts proliferation, HA, and collagen synthesis over a 24-h period following the treatment. The second aim was to assess the reply of a normal fascial fibroblast cell line in vitro with shock waves under different conditions of impulse and energy. After the treatment, we evaluated fascial fibroblasts viability and the expression of HA and collagen types I and III—the main factors in the repair and remodeling process.

## 2. Materials and Methods

### 2.1. Cell Isolation and Culture

Cells were enzymatically isolated from samples of human fascia lata of the thigh (~1 cm × 1 cm size) that were collected from three volunteer patients: two men and one woman, average age 73 ± 5, who were undergoing elective surgical procedures at the Orthopedic Clinic of the University of Padova. The three volunteer patients released written informed consent and all ethical regulations regarding research on human tissues were carefully followed (approval no. 3722/AO/16, study approved on 21 April 2016 by Ethical Committee for clinical trials in the province of Padova). Samples were taken during surgery for partial hip replacement after traumatic femoral neck fractures, from February to April 2021. Fresh samples were put in phosphate buffered saline (PBS) containing 1% penicillin and streptomycin and digested within a few hours of collection. The digestion protocol was carried out by an overnight digestion with collagenase B 0.1% in Hank’s Balanced Salt Solution (HBSS). After centrifuge at 480× *g* for 5 min, the homogenates were transferred in DMEM 1 g/L glucose, 10% FBS, and 1% penicillin–streptomycin antibiotic. Isolated fascial fibroblasts were characterized by immunohistochemical staining with anti-fibroblast surface protein (1B10) antibody (1:100, mouse monoclonal antibodies, AbCam Cambridge, UK), as previously described [32]. Cell cultures were maintained at 37 °C, 95% humidity, and 5% CO_2_ and used from passages 3rd to 9th.

### 2.2. Cell Treatment with ESW

Isolated cells were plated (150 cells/mm^2^ in 500 µL in 24-multiwells containing a glass coverslip) and allowed to attach for 48 h at 37 °C. Before the treatment, the glass coverslip was transferred from 24-multiwells to Falcon^®^ 6-well Tissue Culture-treated plate (3.5 cm of diameter and 2 cm of depth), fixed to the bottom and completely filled with culture medium.

**Figure 1:**
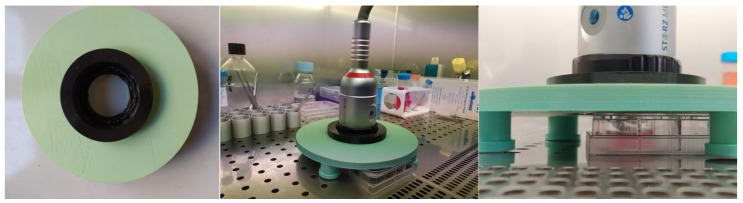
3D-printed ESW cylindrical coil source support in PVC: the support is composed of two separate top parts (black, which adapts to the coil source size) and bottom parts (green, which keeps the support stable and suspended with respect to the 6-wellplates) were designed using Autodesk Fusion 360 (Autodesk, Inc., San Rafael, CA, USA) to minimize the loss of ESW energy at the interface between head of the device and the 6-multiwells.

ESWT was conducted using a Duolith SD-1 T-Top^®^ device (Storz Medical, Tägerwilen, Switzerland) with an electromagnetic cylindrical coil source of focused shock wave (Figure 1), using a 3D-printed support to achieve better focus, keep the probe stable, and fit the probe well to the 6-multiwells.

Briefly, each 6-mutiwell containing cells was placed in vertical alignment with focal area and was adjusted so that the central point of the focal area corresponded to the center of the bottom of the 6-multiwells. For this reason, a 3D-printed support was created for the coil source of ESWs and was kept in contact with and perfectly adherent to the shock unit with the 6-multiwell.

### 2.3. ESW Cylindrical Coil Source Support Design and Fabrication

3D computer-aided design (CAD) models of the ESW cylindrical coil source support were designed using Autodesk Fusion 360 (Autodesk, Inc., San Rafael, CA, USA) to minimize the loss of ESW energy at the interface between head of the device and the 6-multiwells. The CAD data were exported in STL format for the 3D printer. A Ultimaker Creality Ender 3 printer, in its maximum resolution mode, was used for support printing using a polyvinyl chloride (PVC) material. The support printing process took approximately 24 h. The support is composed of two separate top (black, which adapts to the coil source size) and bottom (green, which keeps the support stable and suspended with respect to the 6-wellplates) parts (Figure 1).

**Figure 2:**
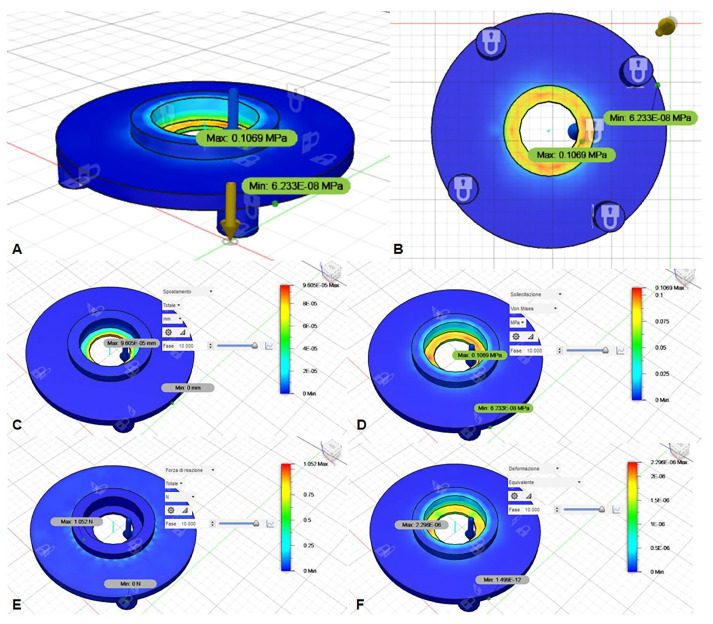
Mechanical simulation of the material used for the support, using Autodesk Fusion 360 (Autodesk, Inc., San Rafael, CA, USA): (**A**) lateral view; (**B**) bottom view; (**C**) shift assessment; (**D**) stress assessment; (**E**) reaction force assessment; (**F**) strain assessment.

Moreover, a mechanical simulation of the material used was conducted using Autodesk Fusion 360 (Autodesk, Inc., San Rafael, CA, USA) to assess the reply of the material to the solicitation given by the coil source and its minimal movement during the delivery of the shots. Results demonstrated a negligible deformation of the black part of the support (Figure 2).

### 2.4. Dose–Response and Time-Course Analysis

To evaluate the best regime to treat the fascial cells, different ESW treatment regimens were investigated. Before, cells were treated at 500 shots, 4 Hz frequency, and an energy flux density (EFD) of 0.45 mJ/mm^2^, leading to immediate death of the cells. After, cells were treated at 100 shots, 2.5 Hz frequency, and an energy flux density (EFD) of 0.25 mJ/mm^2^; the latter was selected as the regimen to use for the ESW treatment. Cells without ESW treatment were used as controls. To evaluate the time-course response of fascial cells to ESW, fibroblasts were fixed at 1, 4, and 24 h, and at the same timing, cells were collected and stained with a 0.4% Trypan Blue solution to evaluate the cytotoxicity by way of Trypan Blue exclusion test. The percentage of viable cells were counted with a TC20^TM^ Automated Cell Counter (Biorad, Milan, Italy). Each experiment was repeated at least thrice in each cell population that was isolated from patients.

### 2.5. Statistical Analysis

Statistical analysis was performed using GraphPad 8.4.2. (GraphPad software Inc., San Diego, CA, USA), and a *p <* 0.05 was always considered as a limit for statistical significance. The normality assessment was carried out using the Kolgomorov–Smirnov test. All results are presented as the mean ± standard deviation. Viability data following different times after SW treatment were analyzed by Kruskal–Wallis test, followed by Dunnett’s test for multiple comparisons to the control (untreated) condition. Differences in the diameter of the vesicles across the different times (1 h, 4 h and 24 h) were statistically analyzed by Kruskal–Wallis test followed by Dunn’s multiple comparisons test for multiple comparisons. The Mann–Withney U test was used for comparisons of the ratio between two times (4 h and 24 h).

### 2.6. Preparation of Samples for Stainings

Isolated cells were plated at a density of 150 cells/mm^2^ in 24 multiwells containing a glass coverslip and were allowed to attach for 48 h at 37 °C; they were then treated with the second ESW regimen (Section 2.3). After monitoring the cells with a Leica DM IL inverted microscope at 1 h, 4 h, and 24 h after the ESW treatment, cells were fixed and 200 µL of 2% paraformaldehyde in PBS at pH 7.4 was added to each well without washing away the treatment solution. This permitted a gentle fixation that could not alter or destroy the vesicles produced by the fibroblasts. After 10 min at room temperature, samples underwent a second fixation (2% paraformaldehyde in PBS, 10 min). After three washings in PBS, samples were stored at 4 °C before the applications of the staining protocols described below. Each experiment was repeated at least three times in each cell population that was isolated from the patients.

### 2.7. Staining Protocols

Fixed cells were stained for 1 h at room temperature with the Alcian blue solution (pH 2.5 for hyaluronic acid, acid mucins, sulfated muco-substances, obtained by 1 g of Alcian blue in 3% acetic acid 100 mL). After a rinse with 3% acetic acid 100 mL (solvent of stain) and two washings in distilled water, the samples were mounted on a glass. The muco-substances took on a stained-blue appearance.

### 2.8. Immunocytochemistry to Detect HABP (Hyaluronic Acid Binding Protein)

**Figure A1:**
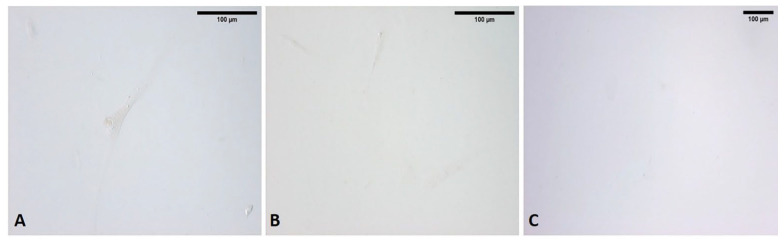
Negative controls with omission: of HABP incubation (**A**); of COL I incubation (**B**); of COL III incubation (**C**).

Endogen peroxidase was blocked with 0.5% H_2_O_2_ in PBS for 10 min at room temperature. After repeated washings in distilled water and a following pre-incubation with a blocking buffer (0.2% bovine serum albumin, BSA, and 0.2% Triton-X, in PBS) for 60 min at room temperature, samples were incubated in hyaluronic acid binding protein (HABP), Bovine Nasal Cartilage, Biotinylated, (Merck Life Science S.r.l., Milano, Italy), diluted 1:1000 in the same pre-incubation buffer, and maintained overnight at 4 °C. After washings, cells were then incubated for 30 min in peroxidase-conjugated streptavidin (Jackson ImmunoResearch Laboratories, Inc., Cambridge House, St. Thomas’ Place, Cambridge, UK), diluted 1:250 in the same pre-incubation buffer. The reaction was developed with 3,3′–diaminobenzidine (Liquid DAB plus substrate Chromogen System kit; Dako). Negative controls were carried out by omitting incubation with HABP, confirming the specificity of the immunocytochemistry analysis with HABP (Appendix A Figure A1A).

### 2.9. Immunocytochemistry to Detect Collagen Type I and III

After blocking of endogen peroxidase by 0.5% H_2_O_2_ in PBS for 10 min at room temperature and repeated washings in PBS, samples were pre-incubated with a blocking buffer (0.1% BSA in PBS) for 60 min at room temperature and then incubated in goat anti-collagen type I, (1:400, SouthernBiotech, Birmingham, AL, USA), or rabbit anti-collagen-III, N-terminal antibody (1:100, Abcam, Cambridge, UK), overnight at 4 °C. After repeated PBS washings, samples were maintained for 1 h in secondary antibody (collagen type I), peroxidase rabbit anti-goat and (collagen III) peroxidase goat anti-rabbit (1:300, Jackson ImmunoResearch Laboratories, Inc.), and then washed in PBS. The reaction was developed with 3,3′–diaminobenzidine (Liquid DAB plus substrate Chromogen System kit; Dako). Negative controls were conducted via omission of the primary antibody, confirming the specificity of the immunostaining (Appendix A Figure A1B,C).

### 2.10. Semithin Sections and Transmission Electron Microscopy (TEM) Analysis

Monolayer cells (24-multiwells) after ESWs treatment (1–4–24 h) were fixed in 2.5% phosphate-buffered glutaraldehyde, post-fixed in 1% osmium tetroxide (OSO_4_), dehydrated in a graded ethanol series, and embedded in epoxy resin (Sigma-Aldrich, St. Louis, MO, USA). Semithin sections (0.5 µm) were stained in 1% Toluidine blue on hot plate for 1 min. Images were acquired with a Leica DMR microscope (Leica Microsystems, Wetzlar, Germany). Ultra-thin sections (60 nm) were cut and collected on 300-mesh copper grids, counterstained with 2% uranyl acetate and Sato’s, and observed in a Hitachi H-300 Transmission Electron Microscope (Electron Microsc. 17:158, 1968) [33].

### 2.11. Image Acquisition and Analysis

Images were acquired with a Leica DMR microscope (Leica Microsystems, Wetzlar, Germany; objectives 10×, 20× and 40× Leica). The ultra-thin sections were examined with a Hitachi H-300 Transmission Electron Microscope. The pictures (enlargement 40×) obtained by immunohistochemistry with anti- HABP were analyzed by conversion to 8-bit, inverted. The diameter of the cytoplasmic and released vesicles (120 vesicles for time) was calculated by ImageJ software (freely available at http://rsb.info.nih.gov/ij/, 5 July 2022). The modal grey values of each vesicle, and of the cytoplasm of its origin cell, were calculated. In the cytoplasm, we calculated the model grey value of at least three different areas of cytoplasm per each cell and calculated the mean. The ratio of each vesicle to the mean of the cytoplasm was then calculated. Finally, the mean ratio with standard deviation was obtained.

## 3. Results

**Figure 3:**
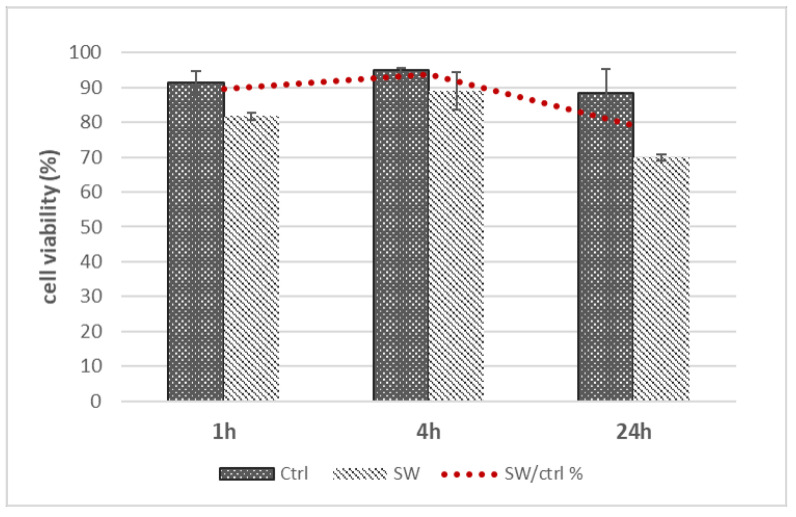
ESWT short-term effects (1, 4 and 24 h) on cell viability of treated fascial fibroblasts compared to untreated; *n* = 9.

The incubation of isolated fascial fibroblasts treated with ESWs did not affect cell viability (Figure 3). ESWs treatment of fascial fibroblasts showed a normal growth pattern, with no statistically significant (*p* > 0.05) difference compared with untreated cells, while being slightly lower than the control cells in all times.

Viability at 1 h decreased to 81.7 ± 1.1% for the treated cells and to 91.3 ± 3.4% for the untreated cells (Figure 3). The same trend of decrease was present at 4 h: 89 ± 5.4% for the treated cells and 95 ± 0.5% for the untreated cells. This was also confirmed at 24 h: 70 ± 0.9% for the treated fascial fibroblasts and 88.5 ± 6.8% for the untreated (Figure 3).

**Figure 4:**
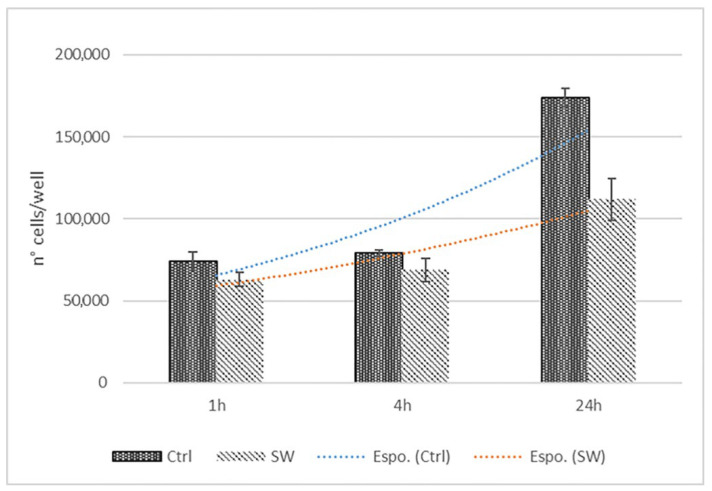
ESWT short-term effects (1, 4 and 24 h) on exponential growth of treated fascial fibroblasts compared to untreated; *n* = 9.

However, even if not statistically significant (*p* > 0.05), the number of cells/well decreased in the treated cells, with few reductions in the cell proliferation (Figure 4).

**Figure 5:**
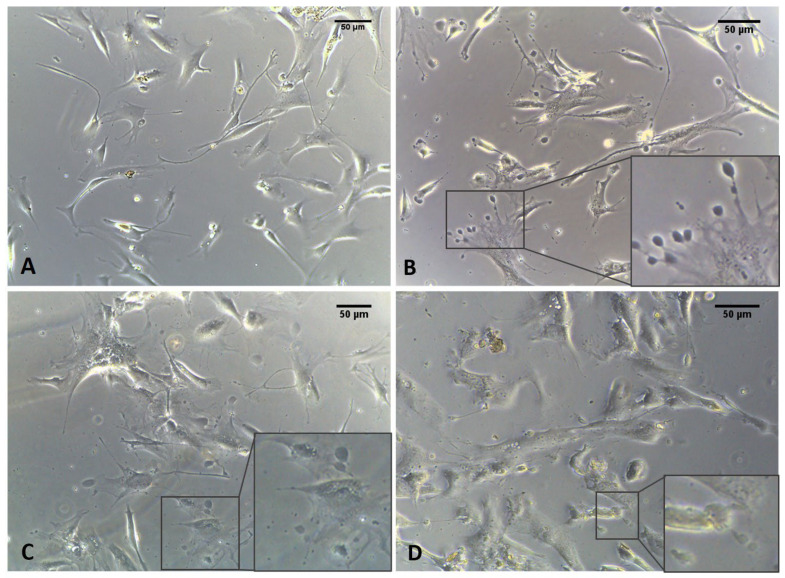
Fascial fibroblasts: control cells (**A**); cells treated with ESWs after 1 h (**B**), after 4 h (**C**), and after 24 h (**D**). The control cells do not show any vesicles (**A**). Only 1 h after ESW treatment (**B**), the formation of cytoplasmic vesicles was observed, becoming especially visible in the cytoplasm of cells after 4 h (**C**), near the nucleus and in the cellular protrusions. After 24 h, the vesicles were also identified (**D**), decreasing those visible inside the cells due to the extrusion and release of their contents into the ECM environment.

Only 1 h after ESW treatment, the formation of cytoplasmic vesicles was observed (Figure 5). In particular, the vesicles were especially visible in the cytoplasm of cells after 4 h (Figure 5), near the nucleus and in the cellular protrusions. Moreover, they were also identified and evident after 24 h while the observation of visible vesicles inside the cells decreased, probably due to extrusion and release of their contents into ECM environment (Figure 5). Furthermore, keeping in view what was highlighted at 4 h, this was the best timing to visualize these vesicles without variation in the cell’s density. Nonetheless, some cells simultaneously showed long- and similar-branched extensions as a sort of protrusion that was rich in vesicles and in non-confluence areas of the culture, changing their morphology (Figure 5).

At that point, after fixation, washing of any vesicles during cell secretion was avoided by adding the fixative solution directly into the well with the medium. In the meantime, the vesicles appeared in different phases, with some in the cell-excretion phase while others, already exocyted, were lost in the fixing solution. No changes in cell density were noted after treatment.

**Figure 6:**
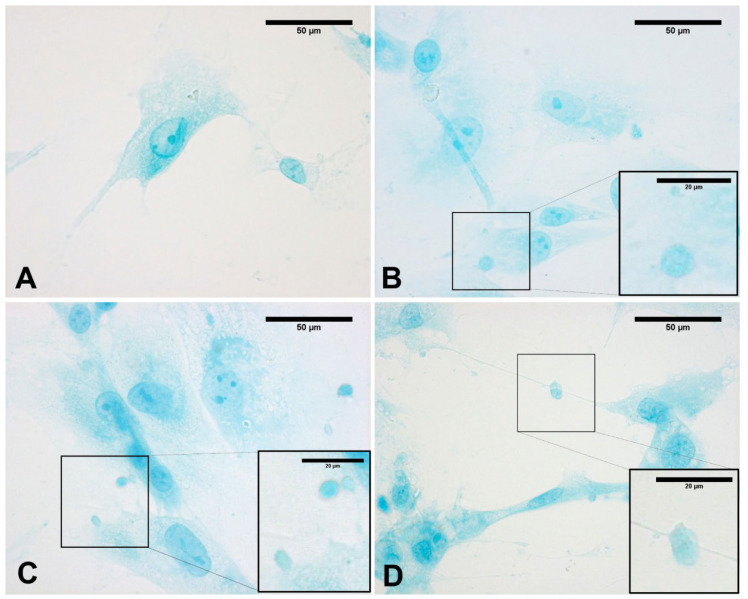
Alcian blue staining: control cells (**A**); cells treated with ESWs after 1 h (**B**), after 4 h (**C**), and after 24 h (**D**). Rectangles: vesicles. In the control cells, no vesicles are present (**A**). The latter are clearly evident within the cytoplasm near the nucleus (**B**); near the membrane, vesicles are in different phases, with some in the cell-excretion phase while others are already exocyted (**C**); vesicles in the cellular protrusions (**D**).

As shown in Figure 6, the content of vesicles in the treated cells as compared to control cells was highlighted by Alcian blue staining, demonstrating the presence and abundance of mucopolysaccharides as confirmed by intense coloring. In the meantime, as is clearly evident in Figure 6, some vesicles near the membrane (probably residues of already exocyted vesicles) were larger and less-intensely stained. In contrast, control cells did not show any vesicles with either staining solution (Figure 6A).

**Figure 7:**
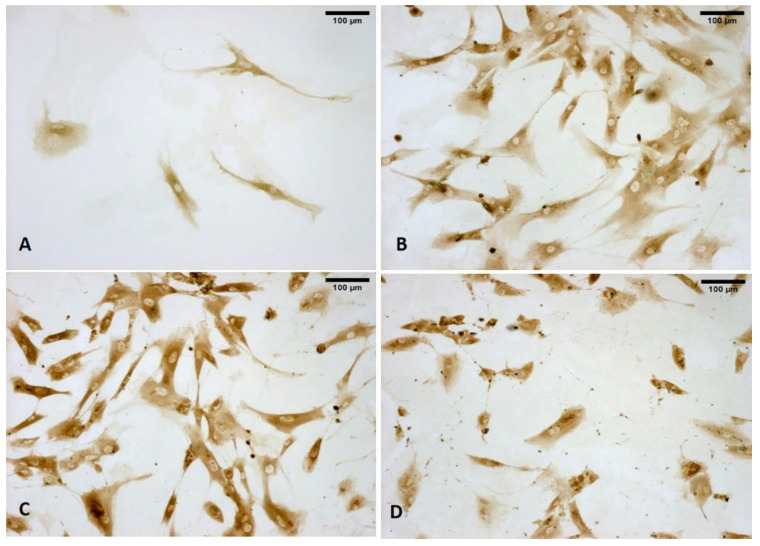
Staining with hyaluronic acid binding protein (HABP): control cells (**A**); cells treated with ESWs after 1 h (**B**), after 4 h (**C**), and after 24 h (**D**). The control cells do not show any vesicles (**A**). The treated samples clearly show HA-rich vesicles of different size and shape in both cytoplasmic and extra-cytoplasmic environments (**B**–**D**).

Moreover, as shown in Figure 7, the vesicles were rich in hyaluronan (HA) at all times, as shown by immunocytochemistry to detect hyaluronic acid binding protein (HABP).

**Figure 8:**
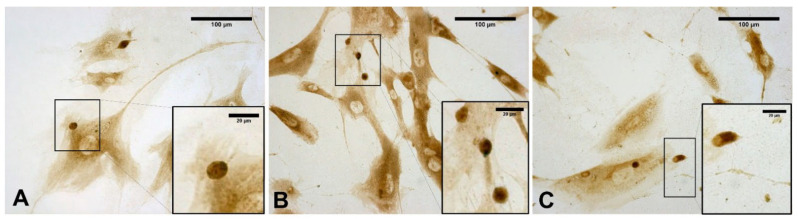
Staining with hyaluronic acid binding protein (HABP): cells treated with ESW after 1 h (**A**), after 4 h (**B**), and after 24 h (**C**). The treated samples clearly show HA-rich vesicles, intensely stained, in both the cytoplasmic and extra-cytoplasmic regions. They are evident in the cytoplasm (**A**); near the membrane, vesicles are in different phases, with some in the cell-excretion phase while others are already exocyted (**B**); exocyted vesicles in the extra-cytoplasmic environment (**C**).

Figure 8 clearly confirms the presence of some vesicles within the cells. At the same time, some vesicles (near the plasma membrane) appeared wider and less-intensely stained (probably because their contents had been exocyted). In contrast, despite staining to detect HABP, control cells did not show any vesicles (Figure 7A).

**Figure 9:**
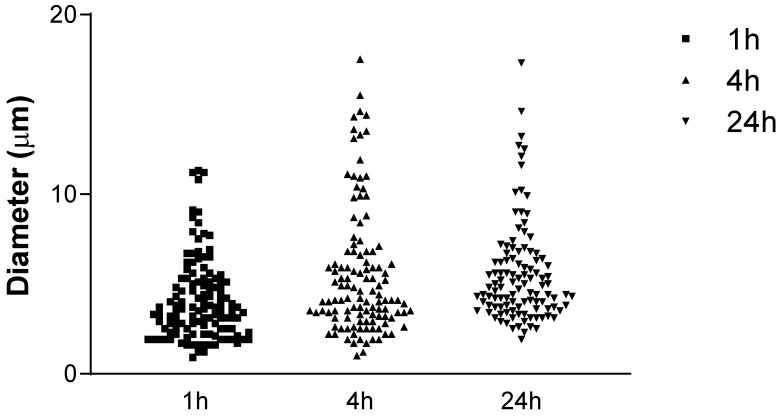
Diameter of the vesicles at different times; h: hours, µm: micrometer.

The observed vesicles in the pictures (enlargement 40×) obtained by immunohistochemistry with anti-HABP had a large size variability, ranging from very small vesicles (diameter: 1–2 µm) to quite large ones (diameter: 10–12 µm). The vesicles at 1 h had a diameter of 4.11 ± 2.30 µm, whereas at 4 h and 24 h, they had, respectively, a diameter of 5.54 ± 3.54 µm and of 5.53 ± 2.8 µm (Figure 9).

In addition, a comparison of vesicle diameter between the different times (1 h, 4 h, and 24 h) is provided in Table 1. According to Dunn’s multiple comparisons test, the comparison between the different times showed a statistically significant difference for 1 h vs. 4 h (*p* < 0.01) and for 1 h vs. 24 h (*p* < 0.0001), whereas it did not show a statistically significant difference for 4 h vs. 24 h (*p* > 0.05).

**Figure 10:**
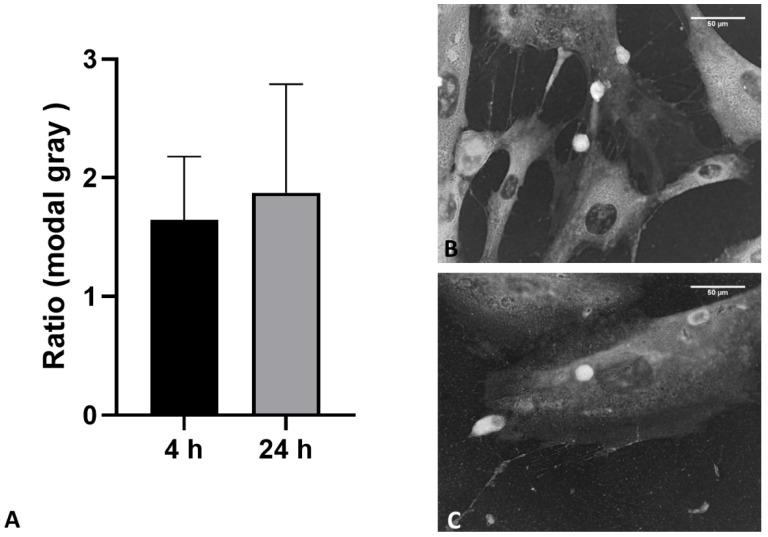
(**A**): Ratio modal gray measurement comparison of each vesicle to the mean of the cytoplasm at 4 h and 24 h. (**B**): 4 h; image showing inverted brightness. (**C**): 24 h; image showing inverted brightness. White: vesicles.

Moreover, the analysis of the modal grey value of each vesicle and of the cytoplasm of its origin cell was for 4 h and 24 h; respectively, 1.64 ± 0.53 and 1.9 ± 0.91 (*p* > 0.05) (Figure 10).

**Figure 11:**
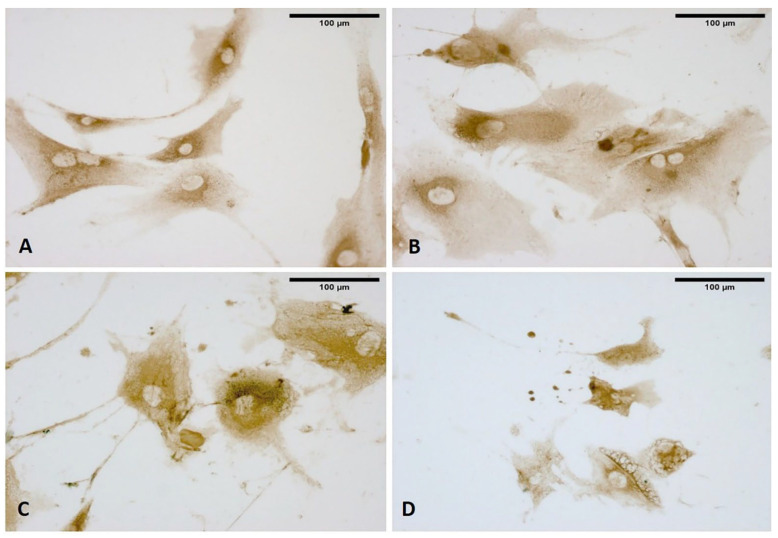
Immunostaining with collagen I (COL I): control cells (**A**); cells treated with ESWs: after 1 h (**B**), after 4 h (**C**), and after 24 h (**D**). The control cells do not show any vesicles (**A**). The treated samples clearly show COLI-rich vesicles in both the cytoplasmic and extra-cytoplasmic environments. Only 1 h after ESW treatment, the formation of cytoplasmic vesicles was observed, especially visible in the cytoplasm (**B**); after 4 h, near the nucleus, near the membrane, and in the cellular protrusions, the vesicles are more evident with different size and shape (**C**); after 24 h, the vesicles were also identified, with a decrease of those visible inside the cells due to the extrusion and release of their contents into the ECM environment (**D**).

The presence of COL I was demonstrated through the use of immunocytochemistry to detect COL I, confirming that the vesicles were also rich in collagen I (Figure 11).

**Figure 12:**
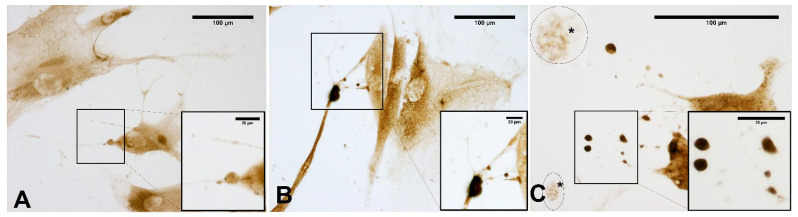
Immunostaining with collagen I (COL I): cells treated with ESWs: after 1 h (**A**), after 4 h (**B**), and after 24 h (**C**). The treated samples clearly show COLI-rich vesicles in both the cytoplasmic and extra-cytoplasmic environments (rectangles) and cellular fragments (circle and asterisk). They are clearly shown and intensely stained in the cellular protrusions (**A**,**B**); exocyted vesicles in the extra-cytoplasmic environment (**C**).

The vesicles respected the same timing as the HA-filled vesicles, with a major release in the first hour that increased over the first four hours and was maintained by 24 h (Figure 12).

**Figure 13:**
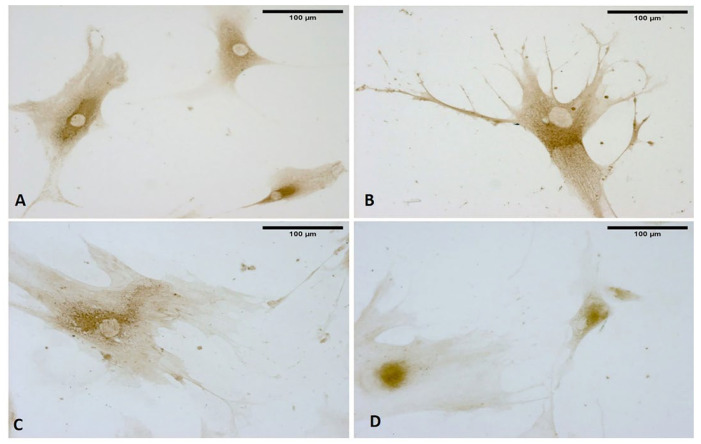
Immunostaining with collagen III (COL III): control cells (**A**); cells treated with ESWs: after 1 h (**B**), after 4 h (**C**), and after 24 h (**D**). The control cells do not show any vesicles (**A**). The treated samples clearly show COLIII-rich vesicles in both the cytoplasmic and extra-cytoplasmic environments. Only 1 h after ESW treatment, the presence of cytoplasmic vesicles is observed in the cytoplasm, near the membrane, and in cellular protrusions, with different size and shape (**B**); after 4 h, near the nucleus, near the membrane, and in the cellular protrusions, the vesicles are more evident with different size and shape (**C**); after 24 h, the vesicles are also identified, with a decrease of those visible inside the cells due to the extrusion and release of their contents into the ECM environment (**D**).

**Figure 14:**
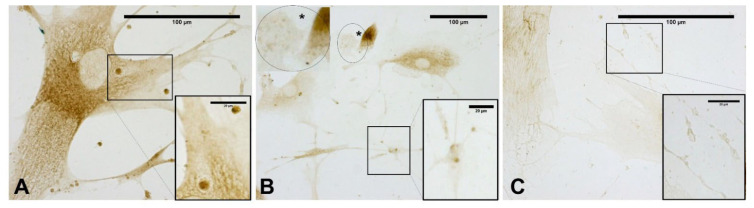
Immunostaining with collagen III (COL III): cells treated with ESWs: after 1 h (**A**), after 4 h (**B**), and after 24 h (**C**). The treated samples clearly show COLIII-rich vesicles in both the cytoplasmic and extra-cytoplasmic environments (rectangles); they are clearly shown and intensely stained inside the cell and in extra-cytoplasmic environment (**A**); in the cellular protrusions, with the presence of cellular fragments (in this image) and a dead cell close to them (circles and asterisk) (**B**); vesicles in the extra-cytoplasmic environment and in cellular protrusions (**C**).

Likewise, to test the presence of COL III, immunocytochemistry was conducted to detect COL III, which confirmed that the vesicles were rich in collagen III (Figure 13), with the same timing of the HA- and COL I-filled vesicles (Figure 14).

Furthermore, the COL I and COL III distribution in the cytoplasm showed a perinuclear staining localized preferentially in the endoplasmic reticulum (Figure 11; Figure 13). As illustrated in Figure 12; Figure 14, collagen I and III secretion of fascial fibroblasts was associated with fascial fibroblasts presenting mainly elongated morphology. Interestingly, the biological significance of this well-known morphological heterogeneity, occurring after the ESW stimulus, is that it is probably able to induce collagen release externally and synthetic activity within the cells (Figure 12; Figure 14), which have already synthesized all their vesicles and wht produce new ones.

Conversely, the HA distribution in the cytoplasm showed a ubiquitous staining of the cell (Figure 7; Figure 8). In particular, as shown in Figure 8, the shape and sizes of these vesicles were also evident in untreated cells, but in the ESW-treated cells, they became more evident, with endoplasmic extensions rich in HA vesicles that were released externally to the cells (Figure 8).

**Figure 15:**
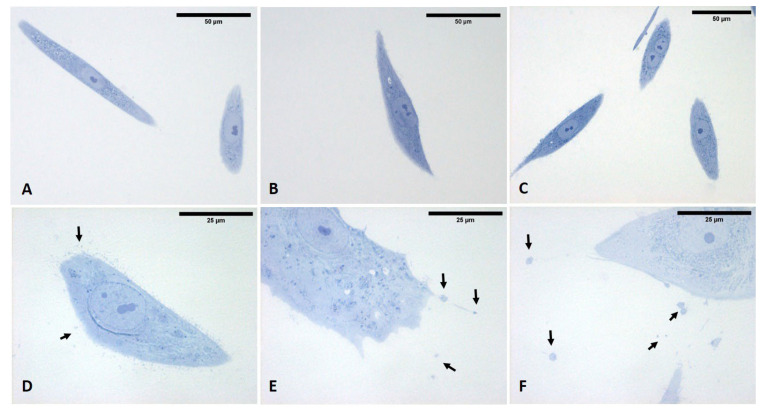
Semithin sections stained with 1% Toluidine blue. (**A**–**C**) control cells, in which vesicles were not present. (**D**–**F**) ESW-treated cells after 1 h (**D**), 4 h (**E**), and 24 h (**F**). Treated cells contained vesicles and amorphous material (pale blue or white). Arrows: vesicles that are shown at high magnifications in Figure 16.

**Figure 16:**
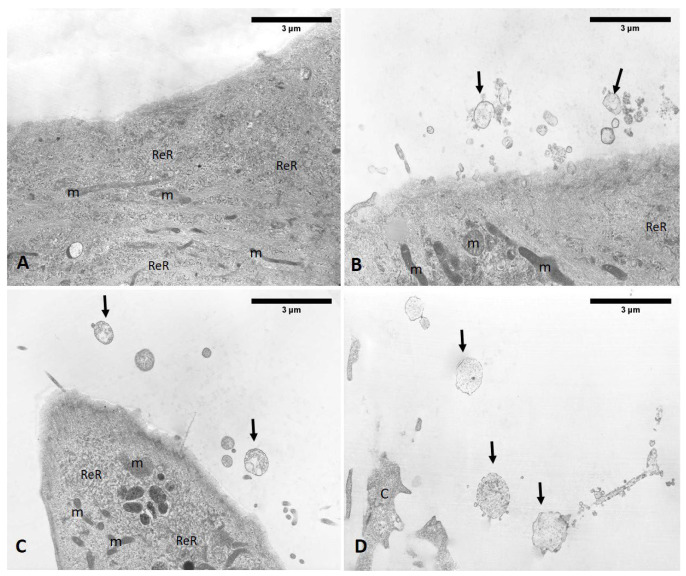
TEM analysis. (**A**) Control cells do not show vesicles. (**B**–**D**) ESW-treated cells after 1 h (**B**), 4 h (**C**), and 24 h (**D**). (**B**) At 1 h, the vesicles are visibly small in size. (**C**) By 4 h, the vesicles increased their diameter; maintained by 24 h (**D**). m: mitochondria; L: lysosomes; ReR: rough endoplasmic reticulum; C: cytoplasm; arrows: vesicles.

The analysis of semithin sections confirmed the presence of material inside the cytoplasm of the ESW-treated cells and confirmed the presence of vesicles with respect to control cells (Figure 15). The latter showed no production of vesicles as confirmed by TEM analysis (Figure 16). Moreover, it was evident by TEM analysis that the fascial fibroblasts treated with ESWs release vesicles in cytoplasm close to the Golgi complex, rough endoplasmic reticulum, and on cytoplasmic extensions of cells or just-excreted vesicles (Figure 16). Finally, it was possible to observe, also via TEM analysis, a large size variability ranging from very small vesicles (diameter: 1–2 µm) to quite large ones (diameter: 10–12 µm). The vesicles showed an increasing trend in dimension, confirming the data on the diameter (Figure 16).

## 4. Discussion

**Figure 17:**
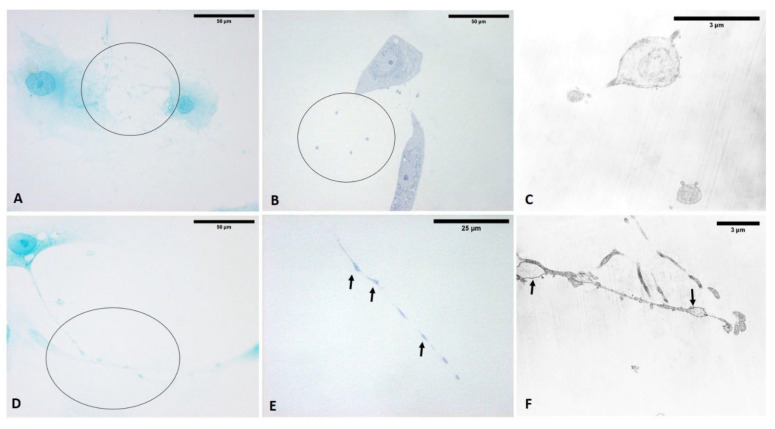
Alcian blue staining (**A**,**D**): vesicles are present in cellular protrusions and in extra-cytoplasmic environment. Semithin sections stained with 1% Toluidine blue (**B**–**E**): vesicles in extra-cytoplasmic environment (**B**) and in cellular protrusions (**E**). TEM analysis (**C**–**F**): the same extra-cytoplasmic vesicles of figure (**C**–**F**), released externally in the extra-cytoplasmic environment, and cytoplasmic extensions of cells with vesicles. Arrows: vesicles; circles: areas in which extra-cytoplasmic vesicles are evident.

The main results of this work provide insight into the possible explanation of ESW-mediated therapeutic advantages in patients affected by myofascial pain (MP). Indeed, for the first time, it was demonstrated that focused ESW treatment can lead to the production of hyaluronan, collagen I, collagen III, and HA-COL I-COL III-rich vesicles in only a few hours in an in vitro culture of fascial fibroblasts. These data are of interest for two reasons: the rapid effect on tissue and the biological effects. Indeed, the metabolic effects of ESWs are usually described after many days, while in the fasciae, they appeared after only one hour. We noted that after only 1 h, the cells seeded in the multi-wells and treated with the ESWs started to produce and release vesicles. Furthermore, after 4 h, in both cytoplasm and particularly in ECM space, vesicles rich in hyaluronan, collagen I, and collagen III were visible. These vesicles were still visible after 24 h, confirming an ECM remodeling process implemented by these cells. Therefore, based on the results of this study, it appears evident that ESWs are able to stimulate fascial remodeling, converting the physical forces into biological activity, and that fibroblasts are mechanosensitive cells [27]. The cavitation effect induced by ESWs into the fascial fibroblasts is probably able to change the membrane potential and to activate molecular autocrine and paracrine signals. It has been hypothesized that ESW mechanical forces, in acting as extracellular information, modulate not only the expression of different genes that regulate function, growth, and differentiation of cells, but also the remodeling of the ECM around the cells, when said forces are transmitted to those cells [34]. The role of extracellular vesicles, as a fundamental signal transmission channel in the central nervous system, is widely reported in the scientific literature [35]. However, it is also widely known that different cell types can release micro-vesicles, and the latter act like mediators of intra-cellular communication, guaranteeing short- and long-range information exchange [36,37], as demonstrated in our previous study [26]. Micro-vesicles are circular membrane fragments that keep the features of the original cell and contain cytosol. Based on their size and molecular composition, they can be differentiated in exosomes with endosomal origin (from 30 to 120 nm), or in synthesizing vesicles (from 100 nm to 1 µm) that rich in phosphatidylserine and proteins associated with membrane lipid rafts [38]. Moreover, it is crucial to distinguish the micro-vesicles secreted in a constitutive way from those secreted following a specific cell activation, such as the vesicles described in this work. The latter are on the order of 1–2 to 10–12 µm, and they are rich in hyaluronan and glycosaminoglycans (Figure 17).

**Figure 18:**
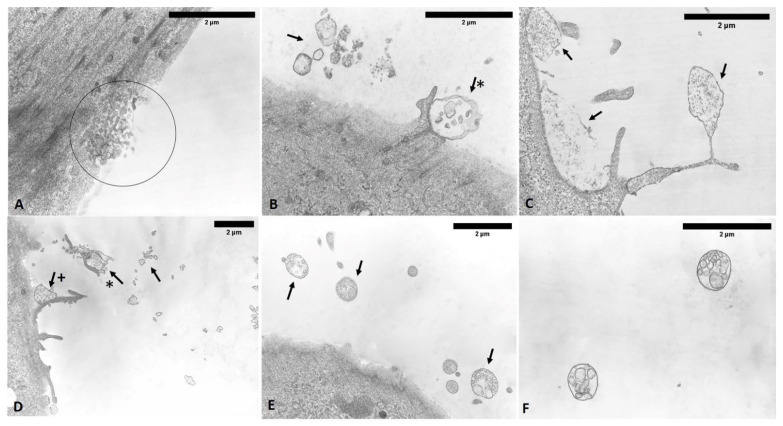
TEM analysis of ESW-treated cells (**A**–**F**). After 1 h, ESW-treated cells showed vesicles with a small size inside the cytoplasm and near the cell membrane, ready for exocytosis (**A**); vesicles increase their diameter in the other times (**B**–**F**). ESW-treated cells show vesicles in extra-cytoplasmic environment and in cytoplasmic extensions. Arrows: vesicles; circles: areas in which vesicles are evident. (**B**): Vesicles of different size and shape (arrows); a large vesicle in exocytosis phase on the border of cell membrane (asterisk with arrow). (**C**) Clear vesicle in cellular protrusion. (**D**) Vesicles of different sizes and shapes, with one vesicle linked to a cell membrane residue (asterisk with arrow) and one in exocytosis (+with arrow). (**E**) Vesicles of different size and shape (arrows) in the extra-cytoplasmic environment. (**F**) Detail of two free vesicles in the extra-cytoplasmic environment with partition walls from aggregation of several vesicles.

In the observed vesicles in the pictures (enlargement 40×) obtained by immuno-histochemistry with anti-HABP, a large size variability appeared evident, ranging from very small vesicles (diameter: 1–2 µm) to quite large ones (diameter: 10–12 µm). The vesicles at 1 h had a diameter of 4.11 ± 2.30 µm, whereas at 4 h and 24 h, they had, respectively, a diameter of 5.54 ± 3.54 µm and of 5.53 ± 2.8 µm. As mentioned before, the vesicles tended to increase in diameter from the 1 h time to the 4 h time, being maintained by the 24 h time. As confirmed from statistical analysis, comparison between the different times showed a statistically significant difference for 1 h vs. 4 h (*p* < 0.01) and for 1 h vs. 24 h (*p* < 0.0001), whereas it did not show a statistically significant difference for 4 h vs. 24 h (*p* > 0.05) (Figure 18). These results provide compelling evidence for immediate effects of in vitro ESW treatment in fascial fibroblasts by 24 h, with effects evident at 1 h that tend to increase over 4 h, remaining over 24 h. The immediate effects noted in this study indicate that the increase in the vesicle dimension gained from ESW treatment may address the regulation, the remodeling, and the formation of extracellular matrix. An in-depth analysis of biochemical features and possible functions of the material contained in the HA-rich vesicles is a task for future investigations [26].

HA is the key component that guarantees the fascial gliding with respect to the all-around structures. In particular, the release of HA into the ECM suggests an increasing of hydration and lubrification of the fascial tissue, which is fundamental for good function of the fasciae. The increase of the secreted hyaluronan can cause a greater, if temporary, fluidity of the tissue, facilitating fascial gliding within and underlying the deep fascia during movement [39].

Our data confirm that ESW treatment promotes and improves the production and release of COL I and COL III, which have a crucial role in the remodeling of connective tissue and, in particular, the fasciae [11]. A pivotal event in the architecture of the fasciae as tendons is collagen fibrillogenesis [11,37]. Both collagen types I and III have been shown to be key players in the regulation of fibril assembly, which follows the row orientation of fascial fibroblasts. Even if the stimulation of collagen production by ESWs has already been described, this study marks the first time that collagen and HA release was highlighted by fascial fibroblasts following ESW treatment. Both of these elements are important for fascial remodeling, as collagen fibers of type I and III are the main constituents of the fibrous component, and HA of the loose component, of fasciae. This study demonstrated that fascial fibroblasts respond to ESWs by synthesizing, remodeling, and regulating the ECM formation, as already reported for other stimuli such as hormones and endocannabinoids [25,26].

We found that ESWs had a dose-dependent destructive effect on fascial fibroblasts in vitro; as such, we chose to treat the cells with the following regimen: 100 shots, 2.5 Hz frequency, and an energy flux density (EFD) of 0.25 mJ/mm^2^. This induced fewer immediate cyto-destructive effects, and there was a better subsequent stimulation of the ECM remodeling but less stimulation on cell proliferation than other fibroblasts from other connective tissues [40,41]. In order to assess the ESW burden on viability and proliferative activity of cultured fascial fibroblasts, a viability assessment was conducted that demonstrated no statistical differences between untreated and treated cells. Consequently, our results indicate that a limited number of ESWs can produce minor damage in soft tissue, favoring ECM remodeling. Since we have immediate effects, within 24 h, by treating fascial fibroblasts, this approach may be appropriate in the treatment of patients with myofascial pain and other muscle-skeletal problems that are indirectly determined by a fascial disfunctions, with modulation of the frequency of ESW treatment sessions as required.

However, some limitations are worth noting. The study only shows qualitative images of treated cells, without a quantification of the production and release of the vesicles. For this type of evaluation, future work should therefore consider the amounts of vesicles inside cells and released in the culture medium over the experimental time-period. Nonetheless, this work constitutes a first step toward understanding the effects of ESW treatment on fascial fibroblasts and the period that immediately follows treatment.

## 5. Conclusions

These findings demonstrate that, in a model of human fascial fibroblasts, ESW treatment can promote ECM remodeling, as well as synthetic activity, over a short time-period, with effects visible by the first hour that are maintained for up to 24 h, suggesting that the clinical benefits and timing of this therapy can be explained by enhanced efficacy in ECM remodeling.

This study indicates that fascial fibroblasts are metabolically “activated” by ESW treatment and significantly induce the synthesis and release of collagen types I and III and HA, compared with untreated cells, in a fast modality. Future studies may shed more light on how different doses of focused ESW treatment, and other types of ESWs, are able to stimulate fascial fibroblasts, thus modulating the therapeutic effect for musculoskeletal dysfunctions and myofascial pain.

## Figures and Tables

**Table 1:** Vesicle diameter measurements comparison between different times (1 h, 4 h, and 24 h). Statistically significant results are showed in bold. **: *p* < 0.01; ****: *p* < 0.0001; n.s: not significant.

Type of Comparison	Mean Rank Diff.	Significant	Summary	*p*-Value
**1 h vs. 4 h**	−43.02	**Yes**	**	**<0.01**
**1 h vs. 24 h**	−61.33	**Yes**	****	**<0.0001**
4 h vs. 24 h	−18.31	No	n.s.	>0.05

## Data Availability

The data presented in this study are available on request from the corresponding author. The data are not publicly available due to privacy.
